# Neurodevelopmental Disorders and Agricultural Pesticide Exposures

**DOI:** 10.1289/ehp.1409124

**Published:** 2015-04-01

**Authors:** Carol J. Burns, Stuart Z. Cohen, Curt Lunchick

**Affiliations:** 1 The Dow Chemical Company, Midland, Michigan, USA; 2 Environmental & Turf Services, Inc., Wheaton, Maryland, USA; 3 Bayer CropScience, Research Triangle Park, North Carolina, USA

We read with interest the analysis by Shelton et al. (2014) of the relationships between maternal proximity to insecticide application and autism spectrum disorders (ASDs) and developmental delay (DD) in children. Although we commend the investigators’ efforts to identify, recruit, and enroll parents of children with ASDs or DD, absent is any confirmation of exposures or that the active ingredients drifted onto the residences or were inhaled or ingested, let alone at dose levels that might be adverse to the fetus (Williams and DeSesso 2014).

The authors noted other sources of potential exposure, including diet and nonagricultural applications that were unmeasured in their assessment. However, there are many factors that reduce the opportunity for participant exposures. Importantly, the inherent properties of each pesticide determine its volatilization and solubility. The method of application and whether the formulation is a liquid or granule also influences drift potential. For example, an orchard air-blast application has a very different exposure potential than a drip-line irrigation application of the same quantity of pesticide to the same crop at the same distance (U.S. EPA 2013a, 2013b). Weather conditions and wind direction influence whether an active ingredient is carried toward or away from a residence (U.S. EPA 2013b). Furthermore, Caldwell and Wolf (2006) found that amounts of ground-spray drift deposited 0.4 km downwind in windy conditions were 0.001% of the applied amounts. Last, being inside, outside, or away from home all factor into human exposures.

Proximity to agricultural pesticide application has not been found to translate to corresponding levels of the pesticide in household dust (Curwin et al. 2005; Fenske et al. 2002; Ward et al. 2006). The California Pesticide Use Registry was evaluated by Nuckols et al. (2007). Although they confirmed agreement of pesticide applications with crop maps, they also recommended biological sampling to validate exposure assumptions for each active ingredient. Correlations of pesticide concentrations in household dust and urinary pesticide metabolite levels in children have been suggested (Lu et al. 2000) but not confirmed (Fenske et al. 2002; Morgan et al. 2008). Several studies of farmers and their families concluded that behavior patterns were more predictive of urinary pesticide concentrations than proximity to the field (Alexander et al. 2006; Arbuckle and Ritter 2005; Thomas et al. 2010).

In their recent review of geographic models in epidemiological studies, Chang et al. (2014) discuss many of these exposure-related issues. The U.S. Environmental Protection Agency has begun to evaluate residential exposures to agricultural pesticides from spray drift and volatilization (U.S. EPA 2014), and there is a growing understanding of off-target drift for each active ingredient. This understanding has permitted the agency to publish a quantitative methodology for assessing residential exposure and risk resulting from spray drift and volatilization of conventional pesticides (U.S. EPA 2014). Risk is the result of the interaction between exposure and toxicity; unfortunately, Shelton et al. (2014) confuse the occurrence of a distant application with exposure. In light of critical weaknesses in exposure characterization in the present case, any relationship between pesticide exposure and the occurrence of ASDs and DD is unknown, and an association between exposure and occurrence is speculation.
